# Endovascular repair of iliac vein perforation during trans-septal mitral valve-in-valve replacement: a case report

**DOI:** 10.1093/ehjcr/ytaf547

**Published:** 2025-10-22

**Authors:** Ting-Wei Kao, Yi-Hsin Hung, Mu-Yang Hsieh, Mao-Shin Lin, Hsien-Li Kao

**Affiliations:** Department of Internal Medicine, National Taiwan University Hospital, No. 7, Chung Shan S. Rd., Zhongzheng Dist., Taipei City 100, Taiwan; Division of Cardiology, Department of Internal Medicine, National Taiwan University Hospital, No. 7, Chung Shan S. Rd., Zhongzheng Dist., Taipei City 100, Taiwan; Department of Internal Medicine, National Taiwan University Cancer Center, No. 57, Alley 155, Section 3, Keelung Road, Da'an District, Taipei City 106, Taiwan; Division of Cardiology, Department of Internal Medicine, National Taiwan University Hospital Hsin-Chu Branch, No. 25, Lane 442, Sec.1, Jingguo Rd., Hsinchu City 300, Taiwan; Division of Cardiology, Department of Internal Medicine, National Taiwan University Hospital, No. 7, Chung Shan S. Rd., Zhongzheng Dist., Taipei City 100, Taiwan; Division of Cardiology, Department of Internal Medicine, National Taiwan University Hospital, No. 7, Chung Shan S. Rd., Zhongzheng Dist., Taipei City 100, Taiwan

**Keywords:** Trans-septal mitral valve-in-valve replacement, Iliac vein perforation, Endovascular repair, Case report

## Abstract

**Background:**

Transcatheter mitral valve-in-valve (TMViV) replacement via trans-septal access is a less invasive alternative for high-risk patients with degenerated bioprosthetic valves. However, life-threatening venous injuries may occur, though they are rarely reported.

**Case summary:**

A 73-year-old man with a prior mitral bioprosthetic valve replacement presented with heart failure due to severe prosthetic stenosis. TMViV was planned via a trans-septal approach. During device advancement through the right iliac vein, a massive venous perforation occurred, resulting in haemorrhagic shock. The cause was likely unrecognized venous calcification. Emergent endovascular repair with covered stents successfully stabilized the patient.

**Discussion:**

This case highlights the risk of catastrophic venous complications during trans-septal TMViV procedures, particularly in patients with venous calcification. Prompt recognition and endovascular intervention can be life-saving in such rare but critical scenarios.

Learning points

- Massive venous injury can occur during large-bore device insertion for structural heart interventions, particularly when unrecognized venous calcification impedes device navigation.
- Bailout use of covered stents is an effective life-saving strategy for managing catastrophic venous perforation.

## Introduction

Mitral valve disease has been recognized as a long-standing clinical burden.^1^ Studies have suggested that 22% of patients with mitral repair and 34% of patients with mitral replacement require subsequent mitral valve replacement (MVR) within 10 years.^2^ Unfortunately, reoperation of failed mitral bioprosthesis is associated with increased morbidity and mortality.^3^ As a result, transcatheter mitral valve-in-valve (TMViV) replacement has emerged as an alternative treatment for patients with high surgical risk.^4^ Research indicates lower in-hospital mortality [odds ratio (OR) 0.64; 95% confidence interval (CI) 0.53–0.78; *P* < 0.01] and reduced major bleeding (OR 0.23; 95% CI 0.10–0.56; *P* = 0.01) in the TMViV replacement group compared with the redo-surgical MVR group, despite a higher comorbidity burden in the TMViV group.^5^

## Summary figure

**Figure:**
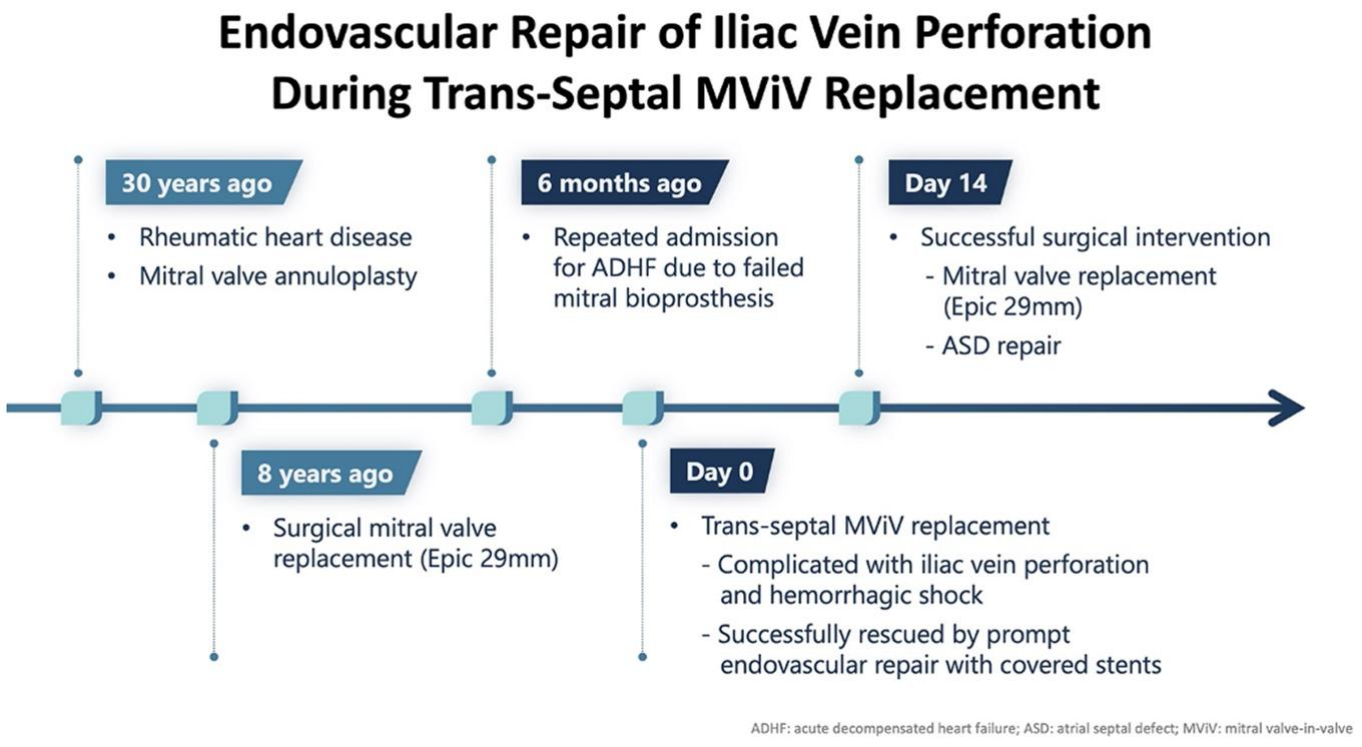


During transcatheter aortic valve replacement, operators commonly exercise caution when advancing sheaths through the femoral and iliac arteries—using stiff guidewires and continuous fluoroscopic guidance to avoid arterial trauma. In contrast, venous access is often approached with fewer precautions. Nevertheless, TMViV procedures can be associated with significant venous complications. In this report, we present a distinct case undergoing trans-septal MViV for failed surgical bioprosthesis. Catastrophic venous perforation occurred during the procedure, likely due to unusual venous wall calcification and mechanical stress from the delivery system. The patient was successfully rescued by prompt endovascular repair.

## Case presentation

A 73-year-old man with a history of rheumatic heart disease underwent mitral valve annuloplasty 30 years ago, followed by MVR with a tissue valve (Epic 29 mm, Abbott Laboratories, USA) 8 years ago. He had repeated admissions for acute decompensated heart failure in the recent 6 months.

Upon admission, he was haemodynamically stable. Physical examination revealed grade III pan-systolic murmur and grade II mid-diastolic murmur at the apex. Biochemistry tests showed creatinine of 1.0 mg/dl and total bilirubin of 2.13 mg/dl. N-terminal pro-brain natriuretic peptide was 1524 pg/ml. The chest X-ray revealed cardiomegaly with lung congestion, and the electrocardiogram showed atrial fibrillation with rapid ventricular heart rate. Echocardiography showed severely dilated left atrium and preserved left ventricle (LV) ejection fraction of 78%. There was a failed mitral bioprosthesis with severe mitral stenosis (mean pressure gradient 14.8 mmHg, mitral valve area by continuity equation 0.6 cm²) and moderate mitral regurgitation.

**Figure 1:**
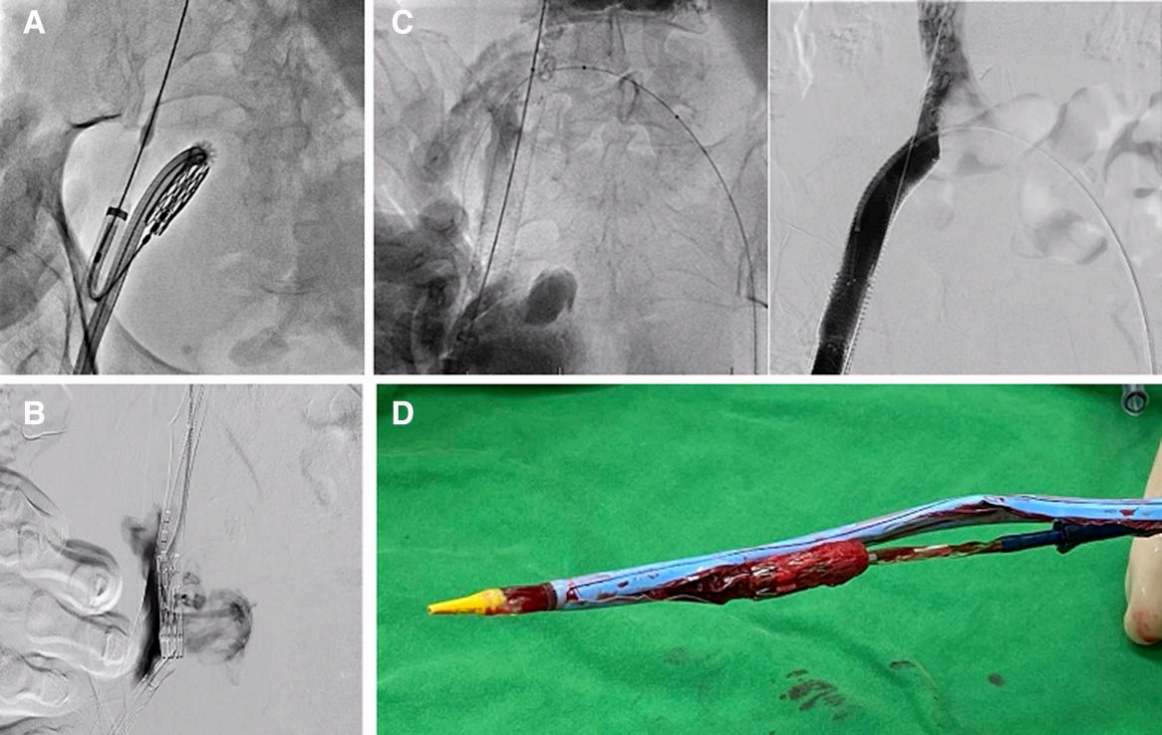
(*A*) Fluoroscopy revealed splitting of the e-Sheath with marked angulation and deformation SAPIEN 3 valve system, suggesting that the device had caused venous perforation. (*B*) Iliac vein perforation. (*C*) Successful repair using covered stents. (*D*) Splitting of the e-Sheath.

Given the prohibitive surgical risk (EuroSCORE II 7.47%, STS Score 9.17%), trans-septal MViV was suggested by the heart team. We planned to implant a 29 mm SAPIEN 3 valve (Edwards Lifesciences, USA) and the preoperative computed tomography (CT) scan showed low risk of LV outflow tract obstruction (neo-LV outflow tract area, 442 mm^2^). The procedure was accessed through the right common femoral vein using a micropuncture set (Cook Medical, Bloomington, USA) under ultrasound guidance. After trans-septal puncture, a 14 Fr e-Sheath was smoothly inserted through the pre-shaped stiff wire. The interatrial septum was then dilated with a 14 mm balloon. However, extreme resistance was encountered while advancing 29 mm SAPIEN 3 Transcatheter Heart Valve (THV) System through the right iliac vein. Suddenly, the resistance dropped, and fluoroscopy revealed splitting of the e-Sheath with the THV system exaggeratedly crooked (*Figure 1A*). Venography confirmed that the patient had a lacerated right iliac vein (*Figure 1B*), resulting in profound haemorrhagic shock with systolic blood pressure dropping from 124 to 76 mmHg.

Continuous blood transfusion was given while manual compression was maintained to control the bleeding. To manage the iliac vein perforation, we performed a contralateral femoral vein puncture and confirmed that the inferior vena cava (IVC) was not involved. The pre-shaped stiff wire remained intact, allowing us to safely remove the deformed device and the e-Sheath. We then re-inserted a new 14 Fr sheath. One Viabahn covered stent (W. L. Gore, USA), 13 × 100 mm, was deployed to repair the right iliac vein (*Figure 1C*). Another two Viabahn covered stents (W. L. Gore, USA), 13 × 100 mm and 13 × 50 mm, were placed to manage femoral vein damage caused while removing the deformed THV system. The bleeding was successfully controlled. The procedure was halted, and the patient was transferred to the intensive care unit.

**Figure 2:**
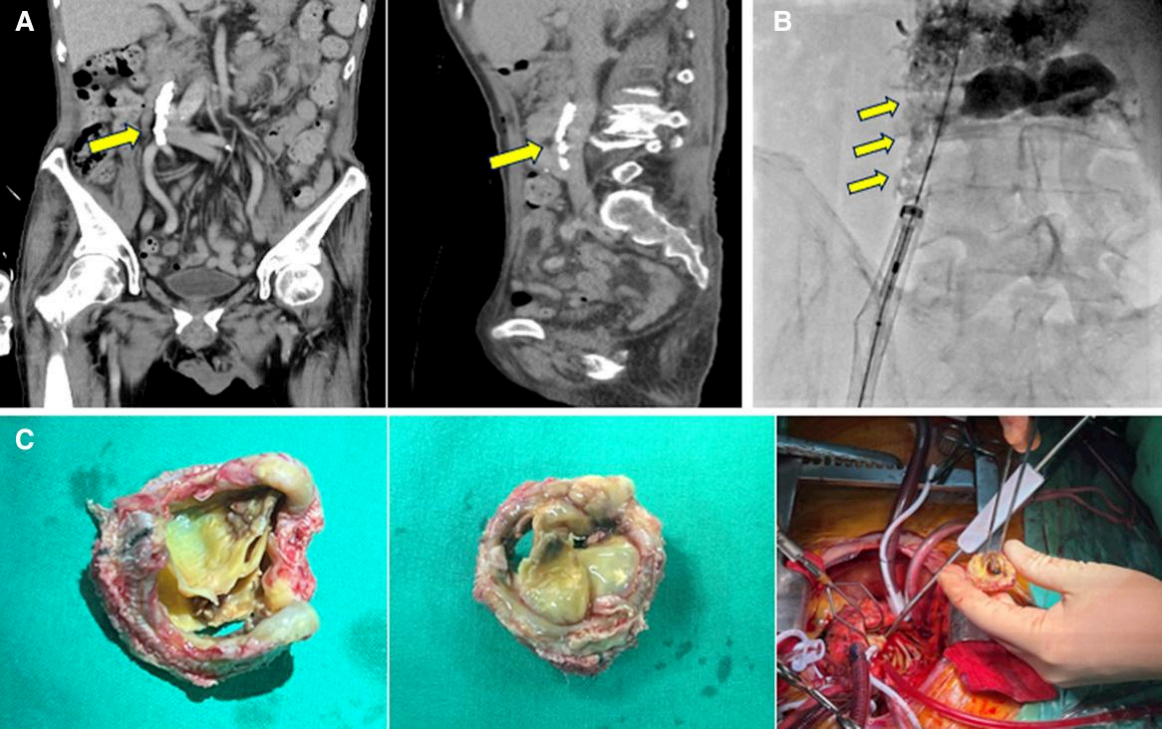
(*A*) The CT scan before transcatheter procedure showed extensive calcification. (*B*) Venous calcification appearing as a ‘broken eggshell’ pattern on fluoroscopic imaging. (*C*) Failed mitral bioprosthesis with pliable anterior leaflet and focal calcification with margin tearing of posterior leaflet.

The e-Sheath was returned to Edwards Lifesciences for evaluation, but no defect was found. Retrospective review of the pre-procedural CT revealed extensive calcification extending from the IVC to the right iliac vein (*Figure 2A*). Fluoroscopic imaging also demonstrated venous calcification, appearing as a ‘broken eggshell’ pattern (*Figure 2B*). The severe and extensive calcification likely contributed to the extreme resistance encountered during valve system delivery, ultimately leading to the catastrophic venous perforation.

Despite initial stabilization, the patient’s condition deteriorated due to residual severe mitral stenosis, mitral regurgitation, and an iatrogenic atrial septal defect (ASD) with left to right shunting, leading to iatrogenic Lutembacher syndrome,^6–9^ along with acute renal failure and liver congestion. Therefore, the heart team suggested surgical intervention on Day 14 following the initial TMViV attempt.

During the surgery, pliable anterior leaflet and focal calcification with margin tearing of posterior leaflet of the mitral bioprosthesis were noted (*Figure 2C*). ASD with 1 cm in diameter was identified at inferior left atrium. Redo MVR with bioprothesis (Epic 29 mm, Abbott Laboratories, USA) was accomplished and ASD was closed with pledgeted suture. Postoperatively, central venous pressure immediately decreased from 27 to 12 cmH_2_O. Both liver and renal function significantly improved. The patient was extubated 2 days later and underwent cardiopulmonary rehabilitation.

## Discussion

The implantation of large-bore devices via trans-septal puncture is increasingly common in structural heart interventions, underscoring the need for meticulous pre-procedural vascular assessment and the capacity for rapid management of venous complications. This case highlights several key considerations. First, the presence of extensive or atypical venous calcification can significantly hinder the advancement of large devices through the venous system. Second, such anatomical challenges heighten the risk of severe venous injury during device delivery, emphasizing the importance of thorough pre-procedural evaluation of venous access. Finally, in the event of life-threatening venous perforation, the prompt use of covered stents provides an effective and minimally invasive salvage strategy.

An extraordinary aspect of this case is the diffuse calcification of the iliac vein, a rare condition typically identified as an incidental finding on imaging studies. While a few cases have been reported in association with underlying conditions such as renal cancer or antiphospholipid syndrome, the aetiology in most instances, including our case, remains unclear.^10^ Possible consequences included pulmonary embolism or deep vein thrombosis.^11–13^ Additionally, severe venous calcification is recognized as a risk factor for vascular complications during transcatheter device delivery. Unfortunately, no widely accepted risk stratification tool currently incorporates venous calcification as a parameter when assessing procedural risk for TMViV. Furthermore, there is a lack of validated scoring systems to guide decision-making between redo-surgical MVR and TMViV in patients with complex vascular anatomy. This highlights a significant gap in current practice and emphasizes the need for individualized pre-procedural assessment in high-risk patients.

Iatrogenic iliac vein perforation during structural heart disease interventions has garnered increasing attention recently. However, literature on this complication remains limited, with only a few case reports available. Landolff *et al*. reported a case of external iliac vein rupture during a left atrial appendage closure. Although the perforation was successfully controlled with two covered stents, the patient required vasopressor support, blood transfusion, and intensive care unit hospitalization.^14^ Yokoyama *et al*. described a case of iliac vein rupture during transcatheter edge-to-edge mitral valve repair, where the patient remained haemodynamically stable, and haemostasis was achieved through overnight balloon occlusion.^15^ More recently, Sava *et al*. reported two cases of iliac vein perforation during ASD and patent foramen ovale closures, respectively, both of which were haemodynamically stable and successfully managed with covered stents as well.^16^ However, to the best of our knowledge, no previous case reports have described life-threatening venous rupture during a TMViV procedure and its subsequent management, as presented in our case. This underscores the potential severity of venous complications in the setting of expanding structural heart interventions and highlights the importance of both comprehensive pre-procedural and peri-procedural evaluation to mitigate risk, as well as the need for effective management strategies to ensure timely rescue of patients.

There are several strategies in pre-procedural planning and intra-procedural decision-making that could help prevent catastrophic venous perforation. In our case, the initial focus of the pre-procedural CT evaluation was primarily on planning for trans-septal access and assessing the risk of left ventricular outflow tract obstruction during TMViV; therefore, dedicated evaluation of the venous access pathway was not emphasized. This experience underscores the importance of thorough venous assessment prior to the procedure, particularly in patients at high risk. If CT imaging of the lower extremities and pelvis is not routinely available for every patient, venous ultrasonography may serve as a practical initial screening tool to identify patients at elevated risk, who may then benefit from further evaluation with contrast-enhanced CT. Second, in our patient, significant calcification appeared as a ‘broken eggshell’ pattern along the right iliac vein on fluoroscopy (*Figure 2B*), while the left iliac vein did not exhibit this finding. This radiographic pattern should serve as a red flag for potential complications. Additionally, when the anatomical structure of the venous access is unclear, venography may be considered to guide procedural planning and avoid adverse outcomes. Lastly, regarding intra-procedural decision-making, the use of a conventional 24 Fr sheath, rather than an expandable e-Sheath, might have facilitated safer navigation through the calcified segment and thereby reduced the risk of venous perforation. Alternatively, accessing the less calcified left iliac vein or employing a trans-apical approach altogether could also be considered viable strategies.

As for the strategies for managing venous rupture, the traditional approach has involved surgical venous ligation or direct repair.^17^ However, more recent treatments have employed covered stents to restore vascular patency.^17^ A review pooling 28 studies demonstrated that the use of covered stents for venous rupture repair is a safe and effective alternative to open surgical repair.^17^ The treatment was technically successful in all cases, with only three perioperative complications reported: immediate stent graft thrombosis managed by AngioJet and IVC filter and asymptomatic pulmonary embolism.^17^ In our case, the emergent placement of covered stents effectively restored vascular patency and prevented further haemorrhage.

To the best of our knowledge, this is the first documented case of iliac vein rupture during trans-septal MViV replacement, offering valuable insights into structural heart disease interventions. This case underscores the importance of preoperative imaging to detect vascular calcification and evaluate procedural risks. Furthermore, the case highlights that immediate endovascular intervention with covered stents can effectively salvage life-threatening venous perforations.

## Lead author biography


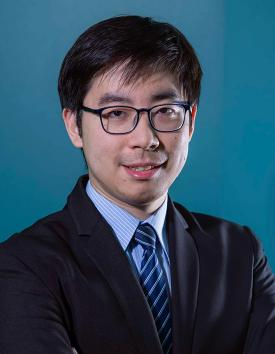


Dr. Ting-Wei Kao is an internal medicine physician with an interest in critical care. During his clinical training, he was involved in the management of the reported patient during the intensive care unit stay. Dr. Kao is committed to developing his clinical skills and expanding his knowledge in the care of critically ill and complex patients.

## Supplementary Material

ytaf547_Supplementary_Data

## Data Availability

All relevant data are included in the manuscript.
