# Supplementary material for: Endovascular repair of iliac vein perforation during trans-septal mitral valve-in-valve replacement: a case report
Source: Eur Heart J Case Rep. 2025 Oct 22;9(11):ytaf547. doi: 10.1093/ehjcr/ytaf547 (PMC12625652; doi:10.1093/ehjcr/ytaf547)

**Supplementary Figure S1.** (A) Trans-esophageal echocardiogram before the transcatheter procedure showed failed mitral bioprothesis with severe mitral stenosis and moderate mitral regurgitation. (B) Trans-thoracic echocardiogram after the failed transcatheter procedure showed iatrogenic atrial septal defect with left to right shunting (subcoastal view).

(A)


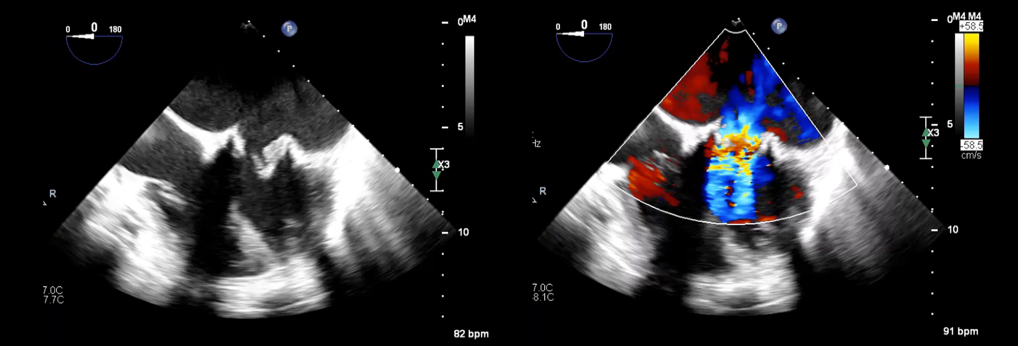

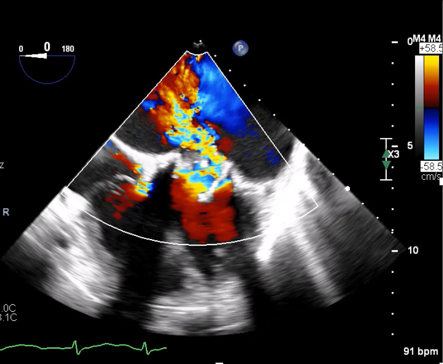

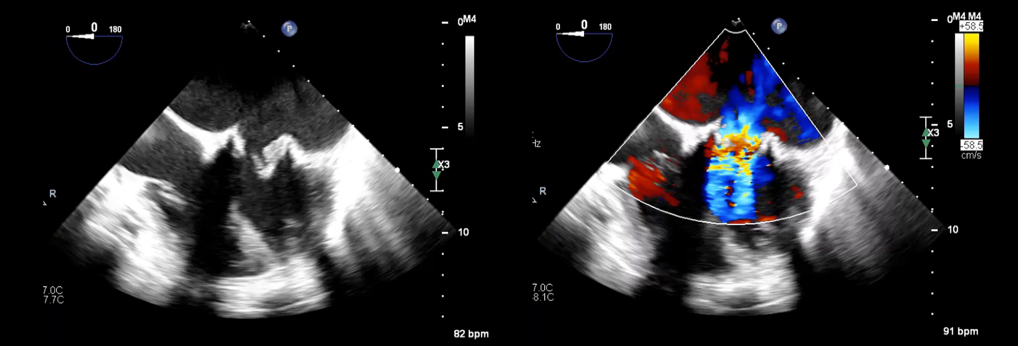


(B)


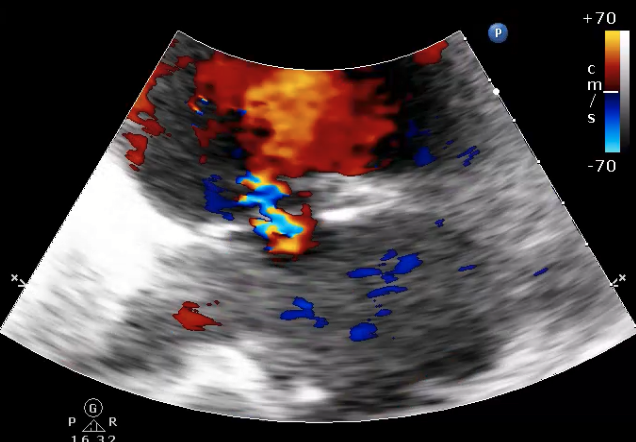

Supplement: ytaf547_Supplementary_Data [file ytaf547_supplementary_data.docx]
